# Research progress on dendritic cell vaccines in cancer immunotherapy

**DOI:** 10.1186/s40164-022-00257-2

**Published:** 2022-01-24

**Authors:** Jifeng Yu, Hao Sun, Weijie Cao, Yongping Song, Zhongxing Jiang

**Affiliations:** 1 The First Affiliated Hospital of Zhengzhou University, Zhengzhou, 450052 Henan China; 2 Henan International Joint Laboratory of Nuclear Protein Gene Regulation, Henan University College of Medicine, Kaifeng, 475004 Henan China; 3 Department of Hematology, The Affiliated Cancer Hospital of Zhengzhou University and Henan Cancer Hospital, Zhengzhou, 450008 Henan China

**Keywords:** Dendritic cell vaccine, Acute myeloid leukemia, Myelodysplastic syndrome, Cancer immunotherapy

## Abstract

Dendritic cell (DC) vaccines induce specific immune responses that can selectively eliminate target cells. In recent years, many studies have been conducted to explore DC vaccination in the treatment of hematological malignancies, including acute myeloid leukemia and myelodysplastic syndromes, as well as other nonleukemia malignancies. There are at least two different strategies that use DCs to promote antitumor immunity: in situ vaccination and canonical vaccination. Monocyte-derived DCs (mo-DCs) and leukemia-derived DCs (DCleu) are the main types of DCs used in vaccines for AML and MDS thus far. Different cancer-related molecules such as peptides, recombinant proteins, apoptotic leukemic cells, whole tumor cells or lysates and DCs/DCleu containing a vaster antigenic repertoire with RNA electroporation, have been used as antigen sources to load DCs. To enhance DC vaccine efficacy, new strategies, such as combination with conventional chemotherapy, monospecific/bispecific antibodies and immune checkpoint-targeting therapies, have been explored. After a decade of trials and tribulations, much progress has been made and much promise has emerged in the field. In this review we summarize the recent advances in DC vaccine immunotherapy for AML/MDS as well as other nonleukemia malignancies.

## Introduction

Acute myeloid leukemia (AML) and myelodysplastic syndrome (MDS) are common hematopoietic diseases characterized by uncontrolled clonal malignant cell proliferation with leukemic blasts replacing the cells that perform normal physiological hematopoiesis and associated symptoms of anemia, bleeding, and infections [1–3]. High-dose induction chemotherapies, followed by allogeneic hematopoietic stem cell transplantation (HSCT), are the only curative options in selected patients [1, 4, 5]. With advances in combined treatment options, the majority of patients can achieve remission after induction chemotherapy; however, only a minority of patients enjoy durable responses. Drug resistance and relapse remain the major challenges, and the 5-year overall survival (OS) rate of AML patients has stagnated at less than 30% [1, 6, 7].

It is well established that the curative effect of allogeneic HSCT derives from allogeneic lymphocyte-mediated graft versus leukemia (GVL) reactivity [8, 9]. However, the complexity, cost and high rates of HSCT related morbidity and mortality have limited the clinical application of HSCT in all patients. Alternative curative approaches to harness antileukemia immunity have been under active investigation in recent decades. Dendritic cell (DC) vaccination as immunotherapy in patients with AML was initiated a few years after DCs were discovered by Ralph Steinman in 1973 [10] but did not see much progress for a long time. Recently, DC vaccination in AML and MDS has received renewed attention with new technologies applied in the vaccine development and patient selections [11, 12]. Different types of DC vaccination strategies using a variety of antigen sources to promote antitumor immunity have been explored. Recently, DC vaccines combined with conventional chemotherapy, systemic monoclonal antibodies or immune checkpoint-targeting strategies, such as those targeting programmed death 1/programmed death-ligand 1 (PD-1/PD-L1), have been studied. Although the development of DC vaccination in patients with AML and MDS has progressed somewhat, there is still a long way to go before therapeutic DC vaccines can be translated from the research laboratory to the bedside. In this review, we summarize the recent advances in DC vaccines immunotherapy for AML/MDS as well as other nonleukemia malignancies.

## DC vaccine cell types and clinical data

**Fig. 1:**
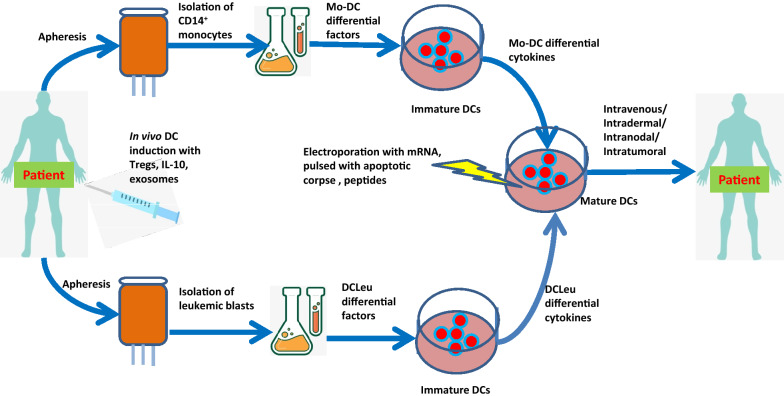
Procedures of DC vaccine preparation for hematological malignancy

DCs are major antigen presenting cells (APCs) that process and present antigens via major histocompatibility complex I and II (MHC I and II) molecules to the innate and adaptive immune systems [13] and play a key role in the interface and crosstalk of the innate and adaptive immune systems [14]. DCs activate NK cells to control pathogens through the innate immune system and activate the adaptive immune system to realize immune memory [15]. Furthermore, DCs form immunological synapses with T cells, resulting in potent T-cell activation against the presented antigens [16]. DCs can enable CD4^+^ T cells to activate B and CD8^+^ T cells, mediate immune memory, and activate Tregs to exert important immunosuppressive functions [17]. Based on developmental, phenotypical and functional criteria, different DC subpopulations have been identified, such as conventional DCs (cDCs), plasmacytoid DCs (pDCs), and monocyte-derived DCs (Mo-DCs) [13]. Based on the automated identification of DCs through unsupervised analysis of conventional flow cytometry and mass cytometry data obtained from multiple species, DCs have been categorized into different classes, and their characteristics and functions have been reported by different groups, as summarized in Table 1 [13, 17–19]. Different types of DC vaccines have been developed in the past years. The major types of DCs used in DC vaccines include Mo-DCs and leukemia-derived DCs (DCleu), which can be modified with different methods. A depiction of DC vaccine preparation procedures and technological advances in hematological malignancy research are summarized in Fig. 1.

**Table 1:** Dendritic cell classification

DC subtype	Morphology	Locations found in vivo	Main distinguishing surface markers detected by flow cytometry		Pattern recognition receptors		Main functions
			Mouse	Human	Mouse	Human	
pDC	Plasma cell-like	Lymphoid tissues and peripheral blood, lung (mouse) and tonsil (human)	CD11clow, MHC-IIlow, B220+, CD317+, SIGLEC-H+, CD172a+, CD209+, CCR2low, CCR9+, CXCR3	CD11c−, HLA-DRlow, CD123+, D303+ (CLEC4C+), CD304+, CCR2+, CXCR3+	TLR7, TLR9, TLR12, RLR, STING, CLEC12A	TLR7, TLR9, RLR, STING, CLEC12A	Control of viral infections, secretion of type I interferon, antigen presentation T-cell priming
cDC1s	Irregular, stellate shape	Lymphoid tissues and peripheral blood	CD11c+, MHC-II+, CD8α+, (resident) CD103+, (migratory) CD24+, XCR1+, CLEC9A+, DEC205+	CD11clow, HLA-DR+, CD141+a, XCR1+, CLEC9A+, DEC205+	TLR2−, TLR4, TLR11−, TLR13, STING, CLEC12A	TLR1, TLR3, TLR6, TLR8, TLR10, STING, CLEC12A	Cellular immunity against tumor and intracellular pathogens, cross-priming
cDC2s	Irregular, stellate shape	Lymphoid tissues and peripheral blood	CD11c+, MHC-II+, CD11b+ (high), CD172a+	CD11c+, HLA-DR+, CD1c+a, CD11b+, CD172a+, CD1a+ (migratory), CD14, CD5 (subset)	TLR1, TLR2, TLR4−, TLR9, TLR13, RLR, NLR, STING, CLEC4A, CLEC6A, CLEC7A, CLEC12A	TLR1−, TLR9, RLR, NLR, STING, CLEC4A, CLEC6A, CLEC7A, CLEC10A, CLEC12A	CD4+ T cell priming
MoDCs	Context dependent	Differentiate from monocytes in peripheral blood, resident in the skin, lung and intestine	CD11c+, MHC-II+, CD11b+, Ly6C+, CD64+, CD206+, CD209+, CD14+, CCR	CD11c+, HLA-DR+, CD1c+, CD11b+, CD14+, CD64+, CD206+, CD209+, CD172a+, CD1a+, CCR2+	Not well defined	Not well defined	Generated by inflammation, CD8+ T cell priming

### Clinical data from monocyte-derived DC vaccines

Mo-DCs are an important DC source for DC vaccinations and can be generated ex vivo from autologous or allogeneic CD14^+^ monocytes [20]. Mo-DCs can be loaded with leukemia-associated antigens (LAAs) [21]. Antigens such as Wilms’ tumor 1 (WT1), preferentially expressed antigen of melanoma (PRAME), and human telomerase reverse transcriptase (hTERT) have been used as LAAs for Mo-DC loading [20, 22]. Mo-DCs can also be loaded with whole apoptotic leukemic cells, leukemia cell lysates or leukemic cell-derived RNA/mRNA [20, 23, 24]. These Mo-DCs can be readministered to AML patients in intradermal or intravenous DC vaccination [20, 22]. A recent feasibility study demonstrated that using cryopreservation of Mo-DCs can be a good method to preserve the cells before use in immunotherapy, avoiding variability within the same individual due to several blood draws [25].

Clinical trials using Mo-DC vaccines for the treatment of AML have demonstrated various successes [20, 24]. In a clinical study, cross-priming of CD8^+^ T cells in vivo by DCs pulsed with autologous apoptotic leukemic cells was used as immunotherapy for elderly patients with acute myeloid leukemia. Antileukemic responses were observed in two of the four study patients with longer periods of disease stabilization [62]. Similarly, vaccinations using Mo-DCs pulsed with leukemic lysates from AML patients who had relapsed after autologous HSCT induced immunological responses and increased autologous T cell ability to stimulate DCs, indicating that Mo-DCs are a feasible cellular therapy for relapsing AML after autologous HSCT [20, 24, 26]. However, more clinical studies are needed to further define the efficacy of Mo-DC vaccines in AML and MDS.

### Clinical data from leukemia-derived DC vaccines

In AML and MDS, DCs can be generated directly from DCleu after culture with different combinations of modifiers [27–29]. Different DCleu-generating protocols have been developed [30]. The morphology of DCleu is similar to that of typical DCs. DCleus have stronger antigen presentation capability, stronger ex vivo antileukemia immune response and enhanced costimulatory molecule expression [12]. The ex vivo production of Mo-DCs and generation of DCleu from leukemic blood cells for vaccinations are challenging processes. However, production protocols have been improved and streamlined [30]. Analysis of these DCleu demonstrated the expression of various specific whole leukemic antigens from patients [31, 32]. The confirmation methods of DCleu include Western blot, immunophenotyping and fluorescence in situ hybridization (FISH) with chromosome-specific DNA probes to detect leukemia-specific numeric or structural chromosomal aberrations in the generated DCleu [33, 34]. Additionally, a special flow cytometric gating strategy has been developed. Using patient-specific blast-staining antibodies together with some unique DC-staining antibodies, some unique antigens that are not expressed on leukemic blasts before DCleu generation can be detected [34, 35]. After blast/DC populations are cultured in DC-generating media, the blast/DC population can be further divided into different subpopulations, such as leukemia-derived DCs, nonleukemia-derived DCs and nonconverted blasts [34]. It has been demonstrated that only mature DCleu can activate immune reactive cells. These mature DCleu express chemokine receptor 7 (CCR7), which is crucial for the migratory capacity of DCleu [12, 36]. Furthermore, mature DCleu also expresses CD83 and secrete IL-12 [37].

Both myeloid leukemia cells and DCs are derived from myeloid progenitor cells, but they have completely different characteristics and T cell stimulation functions. The majority of leukemia cells do not express CD80, only express low levels of CD86 [38, 39] and have poor T cell stimulatory capacity or even induce T cell anergy [40, 41]. Myeloid leukemia cells can be induced to differentiate into DCleu [42] by using various stimulants, such as GM-CSF plus IL-4 with either TNF-α or CD40 ligand (CD40L) [43]. CD40L can induce immature DCleu to become fully mature DCleu and produce stronger T cell stimulation capability [44]. Mature DCleu can gain potent migratory capacity after culture with a group of cytokines [45]. Preliminary reports of clinical trials using AML-DC vaccination in AML patients in the palliative setting have shown therapeutic efficacy [46, 47].

Different loading strategies including loading DCs with select leukemia-derived antigenic peptides [12, 13, 48–50], pulsing DCs with whole leukemia apoptotic bodies [12, 13, 42, 48, 51], pulsing DCs with leukemia lysates [12, 13, 48, 51], and transfecting DCs with mRNA derived from leukemic cells [12, 13, 48, 52], have been explored by different groups. The advantages and disadvantages of these loading strategies are summarized in Table 2.

**Table 2:** Dendritic cell vaccine loading strategies

DC vaccine loading strategies	Advantages	Disadvantages	Main references
Loading DCs with leukemia-derived antigenic peptides	Long-term effect of DC vaccine Broader tumor antigens for desired DC-based vaccines Powerful ability to elicit antigen specific T cell functions Targeting of different epitopes through different DC sources and/or routes of administration	Tumor antigens or HLA molecule expression or both may be lost in the course of disease Tolerance increases due to the expression of shared antigens by normal cells	[12, 13, 48–50]
Pulsing DCs with whole leukemia apoptotic bodies	Contains both known and unknown immunogenic antigens Canbe loaded with costimulatory and adhesion molecules Can activate both the innate and adaptive immune systems to induce tumor-specific CD4 and CD8 T cells	Autoimmunity and/or immunotolerance can be the rare potential issues due to LAAs shared by normal hematopoietic cells	[12, 13, 42, 48, 51]
Pulsing DCs with leukemia cell lysates	Better than apoptotic body vaccines A wider array of antigenic epitopes to stimulate a larger proportion of the CTL repertoire May have interaction of DCs and NK cells	Lower capacity to elicit a broad spectrum of CTLs than apoptotic cells Potential cytotoxicities Longer processing and purification procedures than whole leukemic cell vaccines and mRNA vaccines	[12, 13, 48, 51]
Transfecting DCs with mRNA derived from leukemic cells	Higher transduction efficiency; strong T-cell stimulatory effect Relatively longer mRNA antigen expression time Various leukemic antigens can be included with the total mRNA Amplified total tumor m-RNA can obtain unlimited amount of tumor antigens without the need for the search of specific tumor antigens in each patient	Passive m-RNA loading with weaker stimulatory capacity than m-RNA transfection Safety and vector immunogenicity issues with the viral vectors	[12, 13, 48, 52]

The strategy using genetically modified whole leukemia cells to express costimulatory ligands and/or immunostimulatory cytokines as vaccines has attracted much interest [42]. Genetically modification of leukemia cells to express costimulatory molecules such as CD80 has been successfully applied for almost all leukemia patient samples regardless of the type of leukemia [42]. Preclinical in vitro studies have demonstrated that CD80 transduction enhances the T-cell stimulatory capacity [53] and the immunogenicity of leukemia cells [54, 55].

It has been demonstrated that DCleu increase T-cell activation and shift T cell subsets to a higher activation status in a mixed-lymphocyte culture (MLC), with increased functional T cells and decreased regulatory cells [56, 57]. These findings validate that ex vivo generated DCleu can help to overcome anergy of immune reactive cells in AML and prime effector T cells to exert antileukemic effects against different antigens [31]. In addition, ex vivo-generated DCleu from AML and MDS patients can be used as a predictive factor for the cytolytic potential of leukemia-specific functional T cells induced by DCs. DCleu stimulation can induce specific antileukemic activity against leukemic blasts after MLC [58].

Ex vivo-manufactured DCleu can be readministered to patients as a subcutaneous vaccine after irradiation to avoid transfer of leukemic blasts [34]. Promising results have been reported from several clinical trials with autologous DCleu [46, 47, 59]. Overall, vaccinations with DCleu have been well tolerated, and only a few adverse effects such as extensive eczema, were found in a minority of patients, possibly due to autoimmunity induction [46, 47, 59]. In one leukemia DC vaccine trial, five AML patients received up to four administrations at a biweekly interval without severe adverse side effects. Three of the five patients completed the treatment with four AML-DC vaccinations and remained in a stable condition for 5.5–13 months while the other two patients died from rapidly progressive AML [47]. In a separate trial, five patients received an escalating dose of DCleu once a week after achieving CR from intensive chemotherapy. It was demonstrated that cytotoxic T lymphocytes (CTLs) with IFN-γ-secreting antileukemic activities increased and that WT1-specific CTLs could be detected [46]. After vaccination, specific CD8^+^ T cells and a higher intracellular IFN-γ concentration in CD4^+^ cells were detected [47].

To further improve DC vaccine efficacy, vaccination with both autologous DCs and ex vivo-generated autologous cytokine-induced killer cells (CD3^+^CD56^+^ cells) has also been studied [59]. Compared to patients treated with low-dose chemotherapy alone, patients treated with DCleu vaccination achieved significantly higher complete remission (CR) and partial remission (PR) rates [59].

As mentioned previously, the graft versus leukemia effect is the mechanism underlying the leukemia cure induced by allogeneic HSCT, and the allogeneic DCleu vaccine has attracted researchers’ interest. Using an AML cell line expressing a wide range of LAAs, an allogeneic DCleu vaccine was developed for a phase I clinical trial as a post-HSCT therapy in 12 elderly AML patients. In vivo cellular and humoral immunities were observed with few adverse effects [60]. Patients with no circulating blasts showed unusually prolonged survival whereas patients with circulating blasts died within 6 months. Long-term survival was correlated with maintained T cell levels and induction of multifunctional immune responses [60].

In a phase I/II vaccination clinical trial using a personalized DCleu vaccine with patient-derived AML cells fused with autologous dendritic cells, among the 17 participating AML patients, 12 demonstrated durable expansion of leukemia-specific T cells. Despite a median age of 63 years, 71% of patients remained alive without disease recurrence at a median follow-up of 57 months, demonstrating that this personalized vaccine induces anti-AML cell immunity and provides protection against disease relapse [61]. Currently, on the clinicaltrials.gov website, eight ongoing clinical trials employing either allogeneic DC or autologous DC and different vaccination methods for the treatment of patients with AML and MDS are listed up as of the end of November 2021 (Table 3).

**Table 3:** Ongoing clinical trials of DC vaccines in patients with AML/MDS, melanoma, glioma/glioblastoma, lung cancers, prostate cancer and lymphoma

Type of cancer	NCT ID	Stage of disease	Phase	Source of DC	DC methods	Primary outcomes
AML/MDS	NCT00965224	AML CR with high risk of relapse or previous relapse	II	Autologous DCs	WT1 mRNA-electroporated autologous DCs	Immunogenicity of DC vaccines
	NCT01096602	AML	II	Autologous DCs	DC AML vaccine combined with PD-1 blockade	Toxicity
	NCT01146262	AML first or second CR	I/II	Autologous DCs	Leukemic apoptotic corpse autologous pulsed DCs	Adverse events
	NCT01686334	Relapsed adult non-M3 AML	II	Autologous DCs	WT1 mRNA-electroporated DCs	OS
	NCT03059485	AML at initial diagnosis or first relapse	II	Autologous DCs	DC/AML cell vaccine	PFS
	NCT03291444	Relapsed/refractory leukemia/MDS	I	Autologous DCs	Eps8 peptide-specific DCs	Adverse events
	NCT03679650	AML with allogeneic transplantation	I	Autologous DCs	DC/AML cell fusion vaccine	Fold-increase in AML-specific T cells in the peripheral blood and bone marrow
	NCT03697707	AML in remission with persistent MRD	II	Allogeneic DCs	25E6 cells/vaccination of DCP-001	MRD
Melanoma	NCT00004025	Melanoma (skin)	I/II	Autologous DCs	Autologous dendritic cells transduced with adenoviruses encoding the MART-1 and gp100 melanoma antigens with or without interleukin-2	Safety, dose-limiting toxicity, and maximum tolerated dose
	NCT00017355	Melanoma (skin)	I	Autologous DCs	Autologous DC vaccines made from a patient’s white blood cells mixed with tumor antigens	Safety and tolerability and longevity of melanoma-specific immunity
	NCT00085397	Melanoma (skin)	II	Autologous DCs	Autologous DCs pulsed with gp100 antigen and autologous DCs fused with autologous tumor cells	Immune response
	NCT00126685	Melanoma (skin)	I/II	Autologous DCs	Autologous dendritic cells (DC) transfected with autologous polymerase chain reaction-amplified tumor RNA	Safety, immunogenicity, objective tumor response, time to disease progression, progression-free interval, OS
	NCT00338377	Melanoma	II	Autologous DCs	Lymphodepletion plus adoptive cell transfer with or without DC immunization in patients with metastatic melanoma	Objective response (OR), longitudinal immune response, overall response rate (ORR)
	NCT01082198	Melanoma (skin)	I/II	Autologous DCs	Autologous dendritic cells pulsed with tumor antigen peptides	Immune response, disease-free survival, OS, AEs
	NCT01331915	Melanoma	I/II	Autologous DCs	Proteinic vector targeting DCs coupled to a melanoma antigen,	Safety and toxicity, immune response
	NCT01456104	Melanoma	I	Autologous DCs	Autologous Langerhans-type DCs electroporated with mRNA encoding a tumor-associated antigen	Safety, toxicity
	NCT01753089	Melanoma	I	Autologous DCs	DC activating scaffold incorporating autologous melanoma cell lysate (WDVAX)	Feasibility, safety and biologic activity
	NCT01946373	Melanoma	II	Autologous DCs	Adoptive T cell transfer with or without DC vaccination	Safety
	NCT01973322	Malignant melanoma of skin stage III/IV	II	Autologous DCs	Autologous tumor lysates	Safety, tolerability and feasibility, immune related disease control rate, immunologic efficacy
	NCT01983748	Uveal melanoma	III	Autologous DCs	Adjuvant vaccination with tumor RNA-loaded autologous DCs	Overall survival
	NCT02301611	Melanoma	II	Autologous DCs	Vaccine containing autologous tumor lysate (TL) + yeast cell wall particles (YCWPs) + DCs	Disease-free survival assessment
	NCT02334735	Melanoma	II	Autologous DCs	Mature DC as an adjuvant for NY-ESO-1 and melan-A/MART-1 peptide vaccination	Humoral immune response, cytokine secretion
	NCT02993315	Melanoma (skin)	III	Autologous DCs	Natural DCs pulsed with synthetic peptides	Recurrence-free survival rate
	NCT03092453	Advanced melanoma	I	Autologous DCs	Mature DC vaccination against mutated antigens in patients with advanced melanoma	Immune response of specific T cells
	NCT03325101	Stage IIIA/B cutaneous melanoma	I/II	Autologous DCs	Autologous DCs therapy delivered intratumorally after cryoablation in combination with pembrolizumab	Tumor response rate
	NCT04093323	Refractory melanoma	II	Autologous DCs	Type-1 polarized DC vaccine in combination with tumor-selective chemokine modulation	Objective response rate (ORR)
	NCT04335890	Uveal metastatic melanoma	I	Autologous DCs	Mature DCs loaded with autologous tumor-RNA + RNA encoding defined antigens and driver mutations	Safety, tolerability, dose-limiting toxicities, maximum tolerated dose
Glioma/Glioblastoma	NCT01204684	Glioma, anaplastic astrocytoma, anaplastic astro oligodendroglioma, glioblastoma	II	Autologous DCs	Autologous tumor lysate-pulsed DC vaccination	Time to tumor progression and overall survival
	NCT01291420	Glioblastoma, renal cell carcinoma, sarcomas, breast cancers, malignant mesothelioma, colorectal tumors	I/II	Autologous DCs	Intradermal vaccination with autologous RNA-modified DCs-engineered to express the WT1 protein	Immunogenicity of intradermal DC vaccination
	NCT01567202	Glioma, glioblastoma multiforme	II	Autologous DCs	DCs loaded with glioma stem-like cell-associated antigens against brain glioblastoma multiforme	ORR, PFS, OS
	NCT01808820	Malignant glioma, glioblastoma multiforme, anaplastic astrocytoma, high grade glioma	I	Autologous DCs	Patients derived DC vaccine	Safety and toxicity, AEs
	NCT01957956	Giant cell glioblastoma, glioblastoma, gliosarcoma	I	Autologous DCs	Malignant glioma tumor lysate-pulsed autologous DC vaccine	Safety and toxicity
	NCT02366728	Glioblastoma, grade IV astrocytoma, giant cell glioblastoma, glioblastoma multiforme	II	Autologous DCs	Human CMV pp65-LAMP mRNA-pulsed autologous DCs	Median OS, median PFS
	NCT02465268	Glioblastoma multiforme, malignant glioma, grade IV astrocytoma	II	Autologous DCs	pp65-shLAMP DCs with GM-CSF	Median OS, median PFS
	NCT02771301	Glioma	N/A	Autologous DCs	IDH1R132H-DC vaccine specifically targeting the IDH1R132H mutation	Safety and efficacy
	NCT02772094	Glioblastoma multiforme, glioblastoma	II	Autologous DCs	Autologous DCs loaded with irradiated autologous tumor cells	OS, AEs
	NCT03360708	Giant cell glioblastoma, recurrent glioblastoma, recurrent gliosarcoma	I	Autologous DCs	Malignant glioma tumor lysate-pulsed autologous DC vaccine	Safety and toxicity
	NCT03395587	Glioblastoma	II	Autologous DCs	Tumor lysate-loaded autologous mature DC vaccine	OS, PFS, AEs
	NCT04201873	Recurrent glioblastoma	I	Autologous DCs	Autologous tumor lysate-pulsed DC vaccine	AEs, PFS, OS
	NCT04523688	Glioblastoma	II	Autologous DCs	Autologous DC vaccine loaded with autologous tumor homogenate	AEs, OS
	NCT04552886	Glioblastoma	I	Autologous DCs	TH-1 personalized DC vaccine	Safety and toxicity, Aes
	NCT04963413	Glioblastoma	I	Autologous DCs	Autologous DCs derived from PBMCs loaded with RNA encoding the human CMV matrix protein pp65-flLAMP plus GM-CSF	Proportion of patients for whom CMV pp65 RNA-pulsed DC vaccines can be generated
Lung Cancers	NCT04082182	Metastatic NSCLC	I	Autologous DCs	Intravenous infusion or intradermal injection of MIDRIX4-LUNG DCs, a tetravalent autologous DC vaccine	Toxicity, safety and tolerability, maximal tolerated dose
	NCT04487756	Extensive-stage SCLC	I/II	Autologous DCs	Intradermal injection autologous DC vaccine	PFS, AEs and SAEs (safety)
	NCT04078269	NSCLC	I	Autologous DCs	Novel autologous neoantigen-targeted DC vaccine, MIDRIXNEO-LUNG	Safety and tolerability, toxicity, maximum tolerated and/or feasible dose
	NCT03371485	NSCLC in the advanced and adjuvant settings	I	Allogeneic DCs	Intradermal injection of the allogeneic DC vaccine AST-VAC2 specifically targeting the hTERT protein	AEs
	NCT03546361	AJCC v8 stage IV NSCLC	I	Autologous DCs	Autologous adenovirus CCL21gen-modified DC vaccine	Maximum tolerated dose (MTD)/maximum administered dose (MAD), ORR
	NCT03871205	NSCLC, SCLC	I	Autologous DCs	Personalized autologous neoantigen-loaded DC vaccines	AEs (safety), immunogenicity of neoantigen-primed DC vaccines
	NCT02140996	Epithelial cancers of the lung, breast, ovary, prostate and colon	I	Adenoviral vector vaccine	Ad-sig-hMUC-1/ecdCD40L, adenoviral vector encoding a fusion protein vector vaccine	Safety and tolerability, immunologically active dose level
	NCT03406715	Relapsed SCLC	II	Autologous DCs	Vaccine inclduing autologous DCs with p53 gene insertion (Ad.p53-DC)	Disease control rate (DCR)
Prostate Cancer	NCT00005992	Prostate cancer	I	Autologous DCs	Recombinant prostate-specific membrane antigen (rPSMA)-pulsed autologous DCs (CaPVax)	Safety (AEs)
	NCT01197625	Prostate cancer	I/II	Autologous DCs	Autologous DCs loaded with mRNA from primary prostate cancer tissue, hTERT and survivin	Time to treatment failure defined by two different measurements of PSA levels > 0.5 µg/L with a minimum interval of 4 weeks in patients receiving treatment
	NCT02140996	Epithelial cancers of the lung, breast, ovary, prostate and colon	I	Adenoviral vector vaccine	Ad-sig-hMUC-1/ecdCD40L, adenoviral vector encoding a fusion protein vector vaccine	Safety and tolerability, immunologically active dose level
	NCT02362451	Prostate cancer	II	Autologous DCs	Multiepitope TARP peptide autologous DC vaccine	Difference in rate of PSA change before and after treatment
	NCT02362464	Prostate cancer	II	Autologous DCs	A multiepitope TARP peptide autologous DC vaccine	Safety (AEs)
Lymphoma	NCT01976585	Low-grade B cell lymphoma	I/II	Autologous DCs	Intratumoral injection of rhuFlt3L/CDX-301, Poly-ICLC, and tumor-antigen loaded DCs	Response rate
	NCT03035331	Non-Hodgkin lymphoma	I/II	Autologous DCs	Intratumoral injection of autologous DCs into the cryoablated tumors	Maximum tolerated dose (MTD) proportion of complete responses at maximum tolerated dose (MTD)
	NCT03789097	Non-Hodgkin lymphoma, metastatic breast cancer, and head and neck squamous cell carcinoma	I/II	Flt3L/CDX-301, poly-ICLC	In situ vaccination with Flt3L, radiation, and poly-ICLC	Dose limiting toxicity (DLT)
	NCT00935597	Non-Hodgkin lymphoma, Hodgkin lymphoma, multiple myeloma, chronic lymphocytic leukemia	I	Autologous DCs	Host DC infusion after allo-HSCT	Incidence of severe graft versus host disease (GVHD)

An ex vivo culture system for AML patients simulating physiological conditions has been established using a combination of at least 2 cytokines or response modifiers (“Kits”) to induce DC/DCleu generation [12, 27]. One of the key agents in the “Kits” is IL-15 [62]. In contrast to the conventional IL-4 Mo-DCs used in vaccines, IL-15-differentiated DCs have superior antigen-presenting capability and direct antitumor activity via the expression of IL-15 [63] and make use of both NK cells and γδ T cells in the antitumor immune response [64, 65]. Significantly higher amounts of T effector or T memory cells are found in MLC with Kit-generated DC/DCleu [27, 66]. The clinical application of ex vivo generation of DCs/DCleu may be a reality soon, since all Kit components have been approved for human treatment individually in the clinical setting; for example, GM-CSF is used for neutropenia in patients after chemotherapy or HSCT [67].

### Clinical data from in vivo induced DC vaccines

**Fig. 2:**
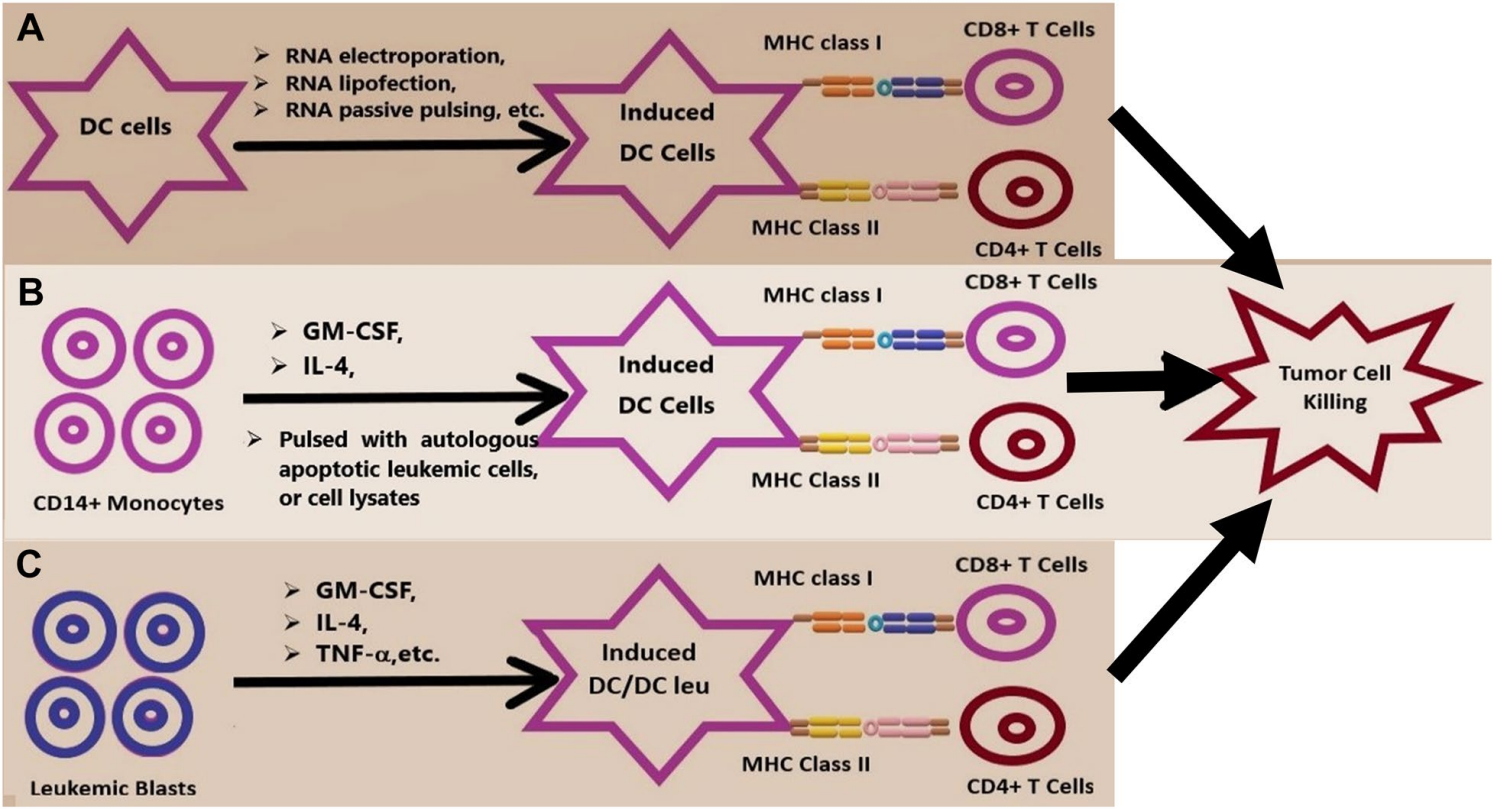
Different types of DC induction: **A** DCs can induced by RNA electroporation, RNA lipofection, passive RNA pulsing etc. **B** Monocyte-derived DCs generated from autologous or allogeneic CD14^+^ monocytes ex vivo can be induced by using GM-CSF and IL-4 and pulsed with autologous apoptotic leukemic cells or cell lysates. **C** Leukemia-derived DCs can be induced ex vivo from leukemic blasts cultured in the presence of different combinations of response modifiers, such as GM-CSF, IL-4, and TNF-α. Subsequently, all these different kinds of DC vaccines can present tumor antigens and costimulatory ligands to T cells. DCs can stimulate both adaptive and innate immune responses against tumor cells, such as acute myeloid leukemia (AML) cells

**Fig. 3:**
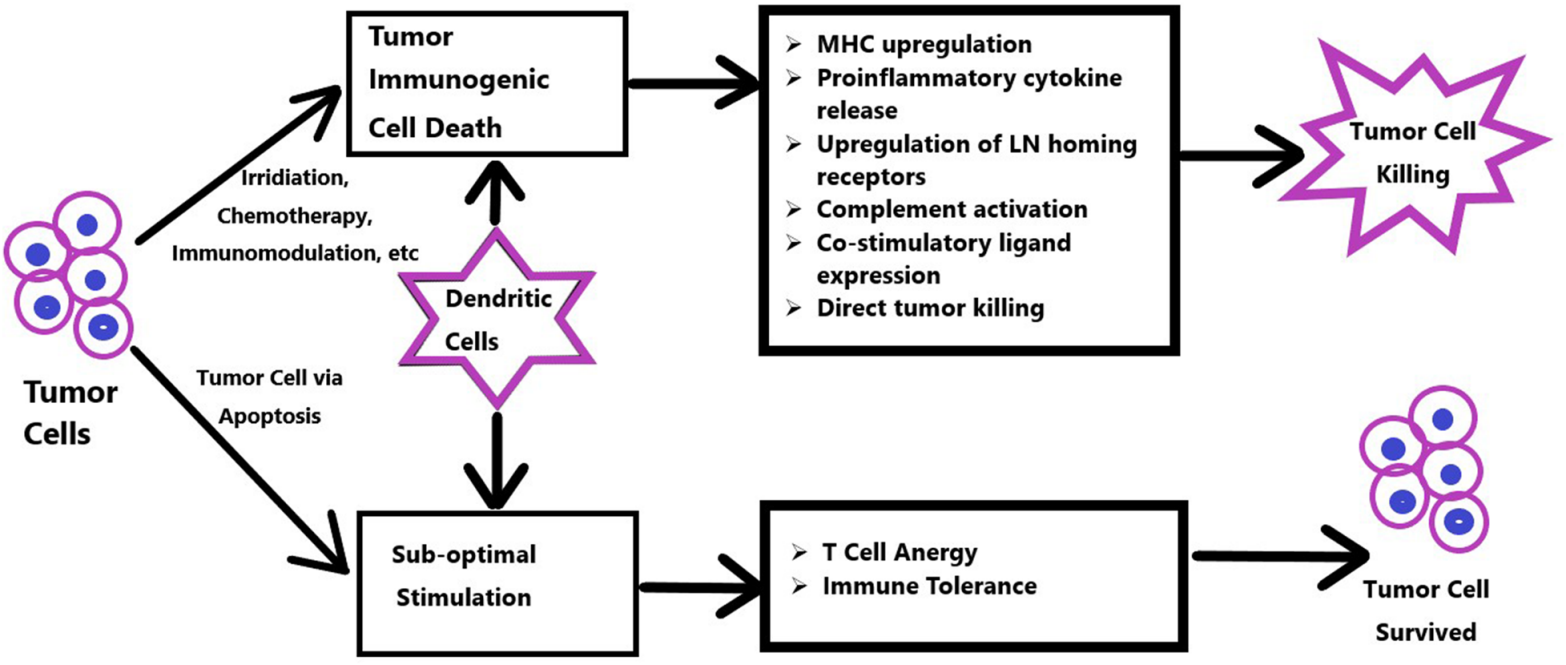
Tumor cells such as AML cells can be killed in different ways, such as irradiation, chemotherapy and immunomodulation. DCs can induce/enhance tumor cell death via different mechanisms, such as MHC upregulation, proinflammatory cytokine release, upregulation of lymph node homing receptors, complement activation, costimulatory ligand expression or direct tumor killing. However, suboptimal stimulation, such as via the apoptosis pathway, can cause T cell anergy or immune tolerance, which allows the tumor cells to survive

Different regulatory mechanisms of in vivo-induced DCs against leukemic cells have been proposed, i.e., regulatory T cells (Tregs), regulatory cytokines (e.g., IL-10) and exosomes [56, 57, 68, 69] (Figs. 2, 3). Exosomes for DC pulsing have demonstrated clinical efficacy in preliminary experiments [70, 71]. Recently, the results of a phase I/II vaccine feasibility study of autologous leukemic apoptotic corpse-pulsed DCs for elderly AML patients in the first or second CR with the pulsed DC administered at doses of 9 × 10^6^ subcutaneously (1 mL) and 1 × 10^6^ intradermally (0.1 mL) were reported. Five doses of vaccine were applied on days + 1, + 7, + 14, + 21, and + 35. No severe adverse events were observed after five DC vaccines were produced for and injected into all five patients in the study [72]. The extended phase II study to delineate the roles of DC-vaccines in AML populations is ongoing under the clinical trial # NCT01146262.

## Strategies for anti-leukemia vaccination

There are at least two different strategies involving DCs that can be used to promote antitumor immunity: in situ vaccination and canonical vaccination. The in situ approach relies on the release of tumor antigens locally through standard therapies to promote DC antigen uptake and tumor immune recognition, whereas the canonical approach relies on the loading of tumor antigens into DCs in vitro before delivering DCs to patients [11]. In recent years, different novel methods for DC vaccine production have been developed, such as immunogenic cell death induction, mRNA transfection, and delivery of peptides to DCs in vivo via cell penetrating peptides [73].

Antigen source selection is crucial in DC vaccination. Previously validated molecules such as peptides, recombinant proteins, whole tumor cells or lysates have been used as antigens in therapeutic vaccine development, although some tumor cell variants may lose antigens through the immune editing process [74]. Either irradiated whole tumor cells or apoptotic whole tumor cells have been used [75]. Whole leukemia cell vaccines can induce immune responses against multiple unknown antigens. However, peptide vaccines elicit immune response against only specific epitopes [76]. The other strategy to generate a whole leukemia cell vaccine is to genetically modify leukemia cells to express costimulatory ligands such as CD80 [76].

Strategies to design AML vaccines include administration of Wilms’ tumor 1 (WT1) peptide with adjuvant [77, 78], DCs with WT1 tumor antigens [79, 80], or DCleu [56]. A systematic review of 9 clinical trials of WT1 peptide vaccines for MDS/AML patients demonstrated that the WT1 peptide vaccine was safe and feasible. Clinical responses and benefits were observed, as some patients achieved and maintained remission for more than 8 years [17]. Mature DCs loaded with RNA encoding WT1 can stimulate an AML-specific T cell-based immune response. Both in vitro and in vivo studies have demonstrated that DCs enhance the induction of tumor-specific immune responses [18]. Clinical trial results show that DC/AML fusion vaccines are well tolerated and yield much less toxicity than other methods; for example, pneumonitis is induced with immune checkpoint inhibitors and cytokine release syndrome is induced with the infusion of activated T cells [81–83]. Significant clinical improvement has been observed with a reduction in peripheral blasts in many patients, with the longest survival being more than 3 years [80, 84–86]. Expansion of the leukemia-specific T cells recognizing WT1, MUC1 and other antigens can enhance immune response specificity. Since MUC1 is only expressed by leukemic stem cells, it has become a unique potential target for this self-renewing population [87]. Numerous studies have demonstrated that DC vaccines can reverse T cell exhaustion and restore T cell function. A preclinical study has demonstrated that targeting the PD-1 and Tim-3 pathways can reverse CD8 T cell exhaustion and enhance ex vivo T cell responses to autologous dendritic/tumor cell vaccines [88]. Furthermore, leukemia derived vaccine can overcome the limitations of checkpoint blockade by evoking clonal T cell responses in a murine AML model. Vaccination with DC/AML fusions resulted in the expansion of tumor-specific lymphocytes and disease eradication in a subset of animals, while the combination of vaccination and checkpoint blockade induced a fully protective tumor-specific immune response in all treated animals. Vaccination followed by checkpoint blockade resulted in upregulation of genes regulating activation and proliferation in memory and effector T cells. Long-term survivors exhibited increased T cell clonal diversity and were resistant to subsequent tumor challenge [89]. A phase I/II clinical trial demonstrated that autologous DCs in combination with chemotherapy can restore the responsiveness of T cells in breast cancer patients [90].

Recently, human γδ T cells have attracted attention in DC vaccine research. Human γδ T cells are heterogeneous subsets of unconventional lymphocytes with human leukocyte antigen (HLA)-unrestricted target cell recognition. Several studies have demonstrated that the cross-talk of γδ T lymphocytes with DCs plays a crucial role in the orchestration of the immune response by bridging innate and adaptive immunity. Studies using a combination of DC-based vaccines with γδ T cells demonstrated strong synergy, long-term tumor control and protection against escaping tumor clones [91].

In WT1 mRNA-electroporated DC vaccination clinical trials in AML patients, the OS rates and the WT1-specific CD8^+^ T cell response were improved significantly [92]. In 30 AML patients with a very high risk of relapse, 13 patients demonstrated an obvious antileukemic response. Nine patients achieved molecular remission and 5 of them sustained this remission after a median follow-up of 109.4 months [92]. Furthermore, patients with different tumors including leukemia, brain tumors, prostate cancer, renal cell carcinoma, pancreatic cancer, as well as HIV infection have been treated with ex vivo-generated mRNA-transfected DCs [93]. In AML and chronic myeloid leukemia (CML) patients, both autologous and allogeneic DCs have been administered as cellular therapy [94]. However, the immunogenicity of leukemia cell vaccines can be limited by many factors [42], and new strategies are urgently needed to induce a potent antileukemic immune response.

## DC vaccines in nonleukemia malignancies

### Melanoma

Melanoma is another malignant tumor in addition to AML and MDS in which DC vaccines have been widely studied. In the past few decades, great progress has been made in the clinical application of DC vaccines loaded with personalized neoantigens, which have been proven to be safe, immunogenic and feasible treatment strategies in patients with melanoma. With the increasing in-depth understanding of DC biology, the next generation of highly efficient cancer vaccines may provide a new immunotherapy strategy for patients with melanoma [95]. Mo-DC vaccines loaded with tumor lysates can affect the tumor microenvironment (TME) and promote the transformation of a “cold” tumor into a “hot” tumor by inducing the activation and infiltration of CD8^+^ T lymphocytes and the upregulation of PD-L1 expression in patients with metastatic melanoma [96]. The combination of a DC vaccine with immune checkpoint inhibitors (ICIs) has been shown to be effective in treatment of melanoma patients. Even after recurrence in patients who received adjuvant DC vaccination, treatment with first- or second-line PD-1 inhibitor monotherapy resulted in a response rate of 52% [97]. A clinical study demonstrated complete and long-lasting clinical responses in patients with immune checkpoint inhibitor-resistant, metastatic melanoma treated with adoptive T cell transfer combined with DC vaccination, with clinical responses induced by tumor-infiltrating lymphocyte (TIL) therapy combined with DC vaccination seen in 4 out of 4 treated metastatic melanoma patients who previously failed ICI therapy [98]. Another study showed that although more patients showed a clinical response to TIL + DC therapy, the combination of TILs and DCs showed no difference in the persistence of MART-1 TILs compared with TIL therapy alone [99]. A study revealed that blockade of inducible costimulatory molecule ligand (ICOSL) on DCs reduced priming of antigen-specific CD8^+^ and CD4^+^ T cells from naïve donors in vitro and that dysregulated NF-κB-dependent ICOSL expression in human DC vaccines impaired T cell responses in patients with melanoma, which supports the implementation of targeted strategies to augment these pathways for improved immunotherapeutic outcomes in patients with cancer [100].

A phase II clinical trial of DC vaccines for patients with stage III/IV melanoma demonstrated improvement in patient survival over the course of a year. However, patients in the DC vaccine treatment group had a higher rate of early local regional relapse than those in the control group, and 80% of patients reported swelling and erythema at the site of intradermal DC-injection [101]. Another study showed that DC vaccines in combination with cisplatin in stage III and IV melanoma patients did not improve clinical outcomes compared to DC vaccination monotherapy [102].

More clinical trials are currently underway. As of the end of November 2021, there were 19 ongoing clinical trials with DC vaccines in patients with melanoma (Table 3). A DC vaccine with natural myeloid DCs loaded with synthetic peptides is currently in a clinical trial for the treatment of stage IIIB and IIIC melanoma patients (NCT02993315). In addition, a personalized vaccine including autologous DCs exposed to autologous whole tumor cell lysate in combination with the chemotherapy drug cyclophosphamide has been explored to treat advanced solid tumor patients with high tumor mutation burden in a phase I clinical trial (NCT03671720).

### Glioma/glioblastoma

As the most frequent and aggressive malignant primary brain tumor, glioblastoma multiforme (GBM) has a highly fatal prognosis and disease recurrence is universal. There is no effective therapy for recurrent disease, and the median survival after relapse is 6.2 months [103]. Animal studies on glioblastoma have demonstrated that DC vaccines can reduce tumor growth, prolong survival, and induce tumor-specific IFN-γ and cytotoxic T-lymphocyte (CTL) responses associated with T cell infiltration of tumors [103]. Numerous clinical trial studies have been initiated in GBM patients and have confirmed the feasibility and safety. Many of these studies reported induction of an antitumoral immune response and indicated improved survival after DC vaccine [104–108]. In a large phase III clinical trial of an autologous DC vaccine in newly diagnosed glioblastoma, the median OS was 23.1 months in patients who underwent surgery and DC vaccination, and vaccination-related grade 3 or 4 AEs were observed in only 2.1% of patients [104]. In another study of vaccination with DCs loaded with TAAs and/or mRNA of neoantigens in combination with low-dose cyclophosphamide in glioma patients, vaccination with DCs loaded with TAAs and the mRNA of neoantigens increased the life expectancy of patients. The median OS was 19 months and no grade 3 or higher AEs were observed [105]. A phase II clinical trial of alpha-type-1 polarized DC-based vaccination in newly diagnosed high-grade glioma revealed a significant survival-prolonging effect in DC-treated glioma patients. Ten of 15 evaluable patients showed positive CTL responses. After 6 years of observation, five patients were still alive, and two of these patients were relapse-free [106]. However, two publications of meta-analyses of randomized controlled studies on the efficacy of DC vaccines for newly diagnosed glioblastoma suggested that dendritic cell vaccines provide no obvious benefits for newly diagnosed glioblastoma [107, 108]. As of the end of November 2021, there were 15 ongoing clinical trials regarding DC vaccines in patients with glioma/glioblastoma (Table 3).

### Lung cancers

Lung cancer is a common malignant tumor that threatens human life and is associated with high morbidity and mortality rates. Calreticulin (CALR) is a characteristic antigen involved in immunogenic cell death in non-small-cell lung cancer (NSCLC). A recent study showed that the CALR-TLR4 complex inhibits NSCLC progression by regulating the migration and maturation of DCs, providing a theoretical basis and ideas for immunotherapy of NSCLC [109]. In a pilot clinical trial study with a personalized neoantigen pulsed DC vaccine for advanced lung cancer (NCT02956551), the objective effectiveness rate was 25%, the disease control rate was 75%, the median progression-free survival (PFS) was 5.5 months, and the median OS was 7.9 months [110]. A randomized-controlled phase II trial of salvage chemotherapy after immunization with a TP53-transfected DC-based vaccine (Ad.p53-DC) in patients with recurrent small-cell lung cancer (SCLC) revealed that the vaccine was safe, with mostly grade 1/2 toxicities and some grade 3 toxicities. The rate of positive immune responses were between 20 and 43.3% in different experimental arms. Although the vaccine failed to improve ORRs to second-line chemotherapy, its safety profile and therapeutic immune potential remain [111]. Small-scale manufacturing of neoantigen-encoding messenger RNA for early-phase clinical trials in lung cancer patients (NCT04078269) has been successfully applied for the clinical evaluation of MIDRIXNEO, a personalized mRNA-loaded dendritic cell vaccine targeting tumor neoantigens [112]. As of the end of November 2021, there were 8 ongoing clinical trials with DC vaccines in patients with lung cancers (Table 3).

Furthermore, a preclinical study on the effect of a DC vaccine loaded with tumor cell lysate (TCL-DCV) on the percentage of CD166^+^ cancer stem cells (CSCs) in the lungs of mice exposed to benzo(a)pyrene (BP) revealed that TCL-DCV reversed the tumorigenic effect of BP in the lungs. Compared to cisplatin, TCL-DCV significantly decreased the percentage of CD166^+^ CSCs, suggesting its potential as a cure for lung cancer [113]. A new PD-1-blocking nanobody (PD-1 Nb20) in combination with tumor-specific DC/tumor cell-fusions augments the broad spectrum of antitumor activity of CD8^+^ T cells, providing an alternative and promising immunotherapeutic strategy for tumor patients who have T cell-dysfunctional or no sensitivity to anti-PD-1 therapy [114].

### Prostate cancer

Patients with high-risk prostate cancer can experience relapse and develop noncurative disease. Vaccines targeting TAAs or tumor-specific antigens have been applied in clinical trials as prostate cancer treatment. Different types of vaccines including DC-based (e.g., sipuleucel-T), peptide/protein-based, or gene-(DNA/RNA) based vaccines have been applied as adjuvant therapy in patients with prostate cancers [115]. Despite the initial success with sipuleucel-T, further DC vaccines have failed to progress. Emerging antigen loading and presentation technologies, such as nanoparticles, antibody-antigen conjugates and virus codelivery systems have been used to improve efficacy [116]. In a phase I trial of an antigen-targeted autologous DC-based vaccine with in vivo activation of inducible CD40 for advanced prostate cancer, immune upregulation and antitumor activity were observed, as were prostate-specific antigen decreases, objective tumor regression and robust efficacy of posttrial therapy [117]. Recently, a long-term first-in-human phase I/II study of an adjuvant DC vaccine in patients with high-risk prostate cancer after radical prostatectomy showed promising results. Among 12 patients with grade 5 prostate cancers, five achieved remission after 84 months, and all mounted immune responses [118]. As of the end of November 2021, there were 5 ongoing clinical trials with DC vaccines in patients with prostate cancers (Table 3).

### Lymphoma

DC vaccine immunotherapy has been used in patients with lymphoma for a long time. The 15-year follow-up of relapsed indolent non-Hodgkin lymphoma patients vaccinated with tumor-loaded DCs demonstrated the absence of toxicity and benefit of active immunization. The results showed that the 5-year and 10-year PFS rates were 55.6% and 33.3%, respectively; 10-year OS rate was 83.3%. Female patients experienced a better PFS and 22% of patients experienced a long-lasting complete response. Different genes including KIT, ATG12, TNFRSF10C, PBK, ITGA2, GATA3, CLU, NCAM1, SYT17 and LTK were differentially expressed in responding tumor patients [119]. The induction of an immune response after allogeneic WT1 DC vaccination and donor lymphocyte infusion (DLI) in patients with hematologic malignancies and posttransplantation relapse demonstrated that vaccines could be successfully produced from samples from all donors. DC vaccination and DLI are well tolerated, and DC vaccines can be used to sensitize the repopulated allogeneic donor immune system to WT1 [120].

In a phase I clinical study in patients with follicular lymphoma (FL), Mo-DCs generated in the presence of IFN-α and GM-CSF (IFN-DC) in combination with low doses of rituximab were administered intranodally. The results indicated that IFN-DCs can synergize with rituximab leading to increased cytotoxicity and T cell tumor infiltration. The overall response rate was 50% and the PET-negative complete response rate was 37%. No grade 3 or higher AEs were observed [121]. As of the end of November 2021, there were 4 ongoing clinical trials with DC vaccines in patients with lymphoma, including intranodally or intratumorally administered vaccines, combined with immunotherapy, radiation or cryosurgery (Table 3).

## Methods for vaccine delivery

A variety of methods to deliver DC-based vaccines to patients have been used, such as intravenous [48, 122, 123], intradermal [123–125] and less frequently intranodal [48, 126] and intratumoral routes [127–130], as well as in vivo DC induction [12] (Table 4). Currently, there is no consensus as to which route of dendritic cell administration is the best for effectively sensitizing T cells. While antigen-loaded DCs can prime T cell immunity regardless of the route, the quality of responses and induction of antigen-specific antibodies may be different depending upon the route of administration [128]. Intravenous administration of antigen-pulsed DCs and subcutaneous administration of immature DCs have been demonstrated to be effective methods for generating sensitized T cells [131, 132]. In a mouse model, local carbon-ion radiotherapy combined with IV DCs augmented the immunogenicity of tumor cells and the maturation of DCs to stimulate antitumor immunity to decrease lung metastases [133]. In a phase I study using a 3 + 3 dose escalation design, the immunogenicity and efficacy of an intravenous DC-targeted liposomal vaccine in twelve patients with metastatic cutaneous melanoma showed that the DC vaccine was well tolerated and did not induce clinically significant toxicity. Partial response and stable disease were observed in one and two patients, respectively [134].

**Table 4:** Routes of dendritic cell vaccine administration

Routes of DC vaccine administration	Advantages	Disadvantages	Main references
Intravenous infusion	Best way for hematological malignancies Can route DC vaccines to nonpreferred areas, e.g., lungs, liver, spleen, bone marrow Delivery of a precise number of DCs to the target T cell compartment	DCs need to go through the blood circulation to reach the tumor sites	[48, 122, 123]
Intradermal injection	Most often used method Administered near superficial lymph nodes May give higher T cell responses than intravenous injection	Allows only 5% of DCs to reach the lymph nodes Efficacy mainly depends on the migratory capacity of DCs to the lymph nodes	[123–125]
Intratumoral injection	Mainly applied in solid tumor patients Produces higher local vaccine concentrations Directly activates infiltrating DCs in the tumor site Easily primes the initial immune response	Considered a traumatic method due to the puncture process	[11, 130]
Intranodal injection	Theoretically, may be the best route since DC migration is not required Superiority over the other routes with regard to sensitization of CD8+ T cells	Extra skills are required to avoid lymph node damage Not commonly used Lack of pulications	[48, 126]
In vivo induction	Administered with Kits. Activate the DCs in vivo Activate both the innate and adaptive immune system and especially leukemia specific T cells followed by an immunoreaction against residual leukemic blasts	Difficult to check the quality and quantity of the DCs May have individual reaction differences More research is needed for validation	[12]

Theoretically, intranodal administration of DCs may be the best route since DC migration is not required. In mouse models, compared to subcutaneous or intravenous immunization, intranodal injection of peptide-pulsed DCs induced significantly greater expansion of antigen-specific T lymphocytes in the spleen and a stronger antigen-specific Th1-type response. Thus, intranodal administration was an effective and feasible method for DC vaccination [94]. It has been reported that intranodal vaccination with semimature DCs primed strong, long-lasting CD4 T cell responses with a Th1-type cytokine profile in advanced melanoma patients [135]. All 5 metastatic melanoma patients in a tumor peptide-based DC vaccination trial developed strong and long-lasting delayed-type hypersensitivity reactivity correlated with the induction of CD4 T cell proliferation in vitro. In vitro stimulation results showed a significant increase in interferon-γ and IL-2 but not IL-4, IL-5, or IL-10 secretion by bulk T cells [135]. Similarly, intranodal administration of adenovirus encoding chimeric CD154 for CML [136], a tolerogenic DC-based vaccine for multiple sclerosis [137] and neoantigen peptide-loaded DC vaccines for ovarian cancer [138] has been reported. The results of a first-in-human phase I trial of intranodal direct injection of adenovirus expressing a chimeric CD154 molecule in fifteen patients with chronic lymphocytic leukemia (CLL) have been reported. The results showed that preliminary clinical responses, including reductions in leukemia cell counts, lymphadenopathy, and splenomegaly were observed. Six patients did not require additional therapy for more than 6 months, and three achieved a partial remission. These results provide rationale for phase II studies in patients with CLL, lymphomas, and CD40-expressing solid tumors [136]. A harmonized study protocol for two phase I clinical trials comparing intradermal and intranodal cell administration has been established, and clinical trials are underway [137]. Another trial demonstrated the clinical and immunological effects of neoantigen peptide-loaded DC-based immunotherapy in a patient with recurrent and chemoresistant ovarian cancer: following four rounds of vaccination with this therapy, CA-125 levels were remarkably decreased, tumor cells in ascites were decreased, and tumor-related symptoms such as respiratory discomfort improved without any adverse reactions [138]. However, studies on intranodal cell administration in AML/MDS patients are lacking.

Intratumoral administration of vaccines has been successfully used in solid tumors, such as breast, ovarian, lung, colorectal and renal cell carcinomas and melanoma. Intratumorally administration of TAAs in combination with immunostimulatory agents was able to activate tumor-infiltrating DCs and induce strong immune responses, resulting in tumor shrinkage or remission [139]. Based on this concept and these research results, TVEC has been approved by the US FDA for clinical use in patients with advanced malignant melanoma. While intratumoral administration of immunostimulatory agents and noncoding RNAs in solid tumors is a plausible method [140] and may remodel tumor metabolism and the immune microenvironment [141, 142], due to the pathogenesis of AML and MDS, intratumoral vaccination to locally activate tumor-infiltrating DCs may not be widely applicable except in the cases with chloroma as the only presentation.

## Enhancing DC vaccine efficacy via coadministration with chemotherapy and checkpoint inhibitors

Hypomethylating agents (HMAs) alter the immune microenvironment in AML. Guadecitabine augments both antigen processing and presentation and increase AML susceptibility to T cell-mediated killing with increases CD4^+^ and CD8^+^ cells targeting syngeneic leukemia cells. Vaccination in conjunction with HMA therapy results in enhanced antileukemia immunity and survival [143]. Furthermore, vaccination with AML cell/DC fusions elicits the expansion of leukemia-specific T cells and protects against disease relapse. The combination of a personalized DC/AML cell fusion vaccine and an HMA demonstrated therapeutic potential [61]. The effects of decitabine on the allogeneic immune reaction were demonstrated in a murine model of DLI significantly greater tumor growth retardation and survival prolongation occurred in mice administered decitabine. Upon prompt DEC and DLI coadministration, DCs were activated, severe GVHD was induced, and survival was significantly decreased compared with DLI alone. The results suggest that DEC primes the allogeneic immune reactions of DLI via DC activation, and GVHD and GVL effects are separable through optimal DLI timing [144].

Studies have revealed that the combination of decitabine or guadecitabine with the NY-ESO-1 vaccine enhances vaccine immunogenicity in AML patients [145, 146]. The de novo expressed NY-ESO-1 protein was naturally processed and presented in a time- and dose-dependent fashion up to 8 days after the start of DAC treatment, and converted the cell lines susceptible to antigen-specific recognition by CD8^+^ T cell clones [145]. T cells from AML patients treated with DC/AML cell fusion vaccine and guadecitabine displayed an increased capacity to lyse AML cells. In vitro studies also demonstrated that decitabine enhances NK cell-mediated cytotoxicity or CD123-specific chimeric antigen receptor T (CAR-T) cell antileukemic activities against AML [146]. In a phase I study, 9 patients with MDS received an HLA-unrestricted NY-ESO-1 vaccine on a nonoverlapping schedule every 4 weeks with standard-dose decitabine. The study demonstrated induction of NY-ESO-1 expression in 7 of 7 patients and NY-ESO-1-specific CD4^+^ and CD8^+^ T-lymphocyte responses in 6 of 7 and 4 of 7 of the vaccinated patients, respectively. Vaccine responses were associated with a detectable population of CD141^hi^ conventional DCs, indicating the potential for induced antigen-directed immunotherapy in MDS patients with limited options [147].

Another strategy to enhance DC vaccine efficacy is to combine DCs with immunomodulators targeting regulatory immune cells to overcome the immunosuppressive mechanisms of leukemia cells [42]. Previous studies have demonstrated that whole leukemia cell vaccines are suppressed by various immunosuppressive mechanisms of leukemia, including B7/cytotoxic T lymphocyte-associated protein 4 (CTLA4) and PD-1/PD-L1, Tregs, and myeloid-derived suppressor cells (MDSCs) [42, 148]. Therefore, blockade of the CTLA4 and PD-1 pathways could be used in combination with whole leukemia cell vaccines [42]. In a recent study, it was observed in a mouse model that T cell exhaustion in Langerhans cell histiocytosis was overcome with PD-1 blockade and targeted MAPK inhibition. The combination of a MAPK inhibitor and anti-PD-1 treatment significantly decreased both CD8^+^ T cells and myeloid Langerhans cells in a synergistic fashion. These results indicate that combined MAPK and checkpoint inhibition is a potential therapeutic strategy [149]. Therapeutic antibodies blocking the PD-1 pathway have been widely used in solid tumors [150]. DC/PD-1 immunotherapy combinations are currently under preclinical and clinical investigation in recurrent advanced brain tumors, advanced and relapsed NSCLC, MM and advanced renal cell carcinoma [151]. Additionally, various other combination therapies that exploit alternative immune targets and other therapeutic modalities have been explored for cancer treatment [152–154], especially with the development of a new generation of immune checkpoint inhibitors and other inhibitory targets [155–161]. The combination of a DC/AML cell fusion vaccine and checkpoint blocking therapy provides unique synergy to induce durable activation of leukemia specific immunity, protect against lethal tumor challenges, and selectively amplify tumor-reactive clones [162]. Different combinations designed to activate the endogenous T cell response through checkpoint blockade appear suitable and are being increasingly tested [163].

In some nonhematological malignancies, DC vaccines have shown an increase in the survival rate of patients in the late stage of tumor disease and have had a significant impact on the destruction of distant metastases. The combination of DC vaccines with another immunotherapy or traditional anticancer methods can be used in treating patients in the early stage of disease or preventing recurrence and metastasis [164].

Combining cancer vaccines with immunomodulatory drugs is currently regarded as a highly promising approach for boosting tumor-specific T cell immunity and eradicating residual malignant cells. Recently, a study of the FL mouse model with a new vaccine including IFN-DCs loaded with apoptotic lymphoma cells demonstrated that lenalidomide improves the therapeutic effect of an interferon-α-dendritic cell-based lymphoma vaccine, which may lead to evaluation of the combination in clinical applications [165].

## Challenges of DC vaccines

Although much progresses has been made in the field of DC vaccines, there are several challenges to the wider application of leukemic DC vaccines. In previous trials, failure to generate sufficient numbers of qualified AML-DCs was the most common reason for excluding patients from the study [46]. The high cost of the stimulants required to differentiate leukemic DCs was another challenge [42].

A critical lesson learned, however, is the insufficient therapeutic efficacy of vaccination using genetically modified GM-CSF-secreting leukemia DCs, which is probably due to the immunosuppressive effects of GM-CSF [166]. The lack of immunogenicity of the whole leukemia cell vaccine may be due to the immunosuppressive effect of phosphatidylserine (PS) exposure to inactivated immune responsive T cells [167]. However, studies have shown that inhibition of the PS recognition process increases the immunogenicity of irradiated lymphoma cells, suggesting that endogenous adjuvants combined with dying tumor cells can be used to target tumor immune rejection [168].

Immunosuppressive factors from malignant cells can impede the functions of both DCs and T cells and hinder the vaccine-generated protective immune response. Defects in hematopoietic progenitor cells and an abnormal bone marrow niche render hematopoiesis seriously ineffective in leukemia patients. These factors bring additional challenges and accentuate systemic immunosuppression and DC malfunction [66]. Overall, the objective response rate (ORR) of DC vaccine was reported to be approximately 15% [122]; its therapeutic efficacy has to be improved.

Different mechanisms of weak immunogenicity of DCs have been reported, including failure to induce leukemia-specific CTLs [49], failure to activate NK cells or γδ T cells [65, 169, 170], failure to overcome the immunosuppressive action of Tregs and MDSCs [171], and undesired immune effects from Tregs and MDSCs [172]. Therefore, novel strategies, including DC vaccines combined with HMAs [146], NK cell infusion or immune checkpoint blockade therapy in relapsed/refractory AML and high-risk MDS patients need to be further validated.

## Conclusions and future directions

Many difficulties remain and prevent the widespread application of DC vaccine immunotherapy. These limitations include weak cellular immune responses, high costs and time-consuming processes [13]. Despite the limited clinical efficacy, DC vaccinations still constitute a promising new strategy for AML and MDS treatment, as well as the treatment of other malignancies [13, 66, 164] with the validated safety and feasibility [62]. In addition to DC vaccination combined with systemic monoclonal antibody and immune checkpoint blockades, incorporation of PD-1/PD-L signaling into DCs can enhance DC mediated activation of T/NK cells and prevent Tregs stimulation [150, 173]. With the increasing clinical efficacy and application of DC-based vaccine therapy in patients with solid tumors [174], there is increasing interest in combining DC vaccines with conventional therapy, such as HMAs in the frontline treatment of elderly AML patients [108]. A recent study demonstrated that transgelin-2 is an essential protein for cancer and immunity and can act as a double-edged sword for cancer therapy. Engineering and clinical application of this protein may lead to a new era of DC-based cancer immunotherapy [175]. We believe that use of a carefully designed personalized DC vaccine in combination with other appropriate treatment strategies may constitute a valuable option for future treatment of patients with AML/MDS and other malignancies.

## Data Availability

All clinical trials related information was obtained from public databases.

## Abbreviations

Allo-HSCT: Allogeneic hematopoietic stem cell transplantation
AML: Acute myeloid leukemia
APC: Antigen presenting cells
Apo-DC: Apoptotic CLL cells (Apo-DC)
BDC: Blood dendritic cells
CALR: Calreticulin
CAR-T: Chimeric antigen receptor T cells
CCR7: Express chemokine receptor 7
cDCs: Conventional DCs
CLL: Chronic lymphocytic leukemia
CR: Complete remission
CR: Complete remission
CTL: Cytotoxic T-lymphocyte
CTLA4: Cytotoxic T-lymphocyte-associated protein 4
Dcleu: Leukemia-derived dendritic cells
DCs: Dendritic cells
DLI: Donor lymphocyte infusion
FISH: Fluorescence in situ hybridization
FL: Follicular lymphoma
GBM: Glioblastoma multiforme
GMP: Good Manufacturing Practice
GVL: Graft versus leukemia
HMA: Hypomethylating agents
HSCT: Hematopoietic stem cell transplantation
hTERT: Human telomerase reverse transcriptase
ICI: Immune checkpoint inhibitor
ICOSL: Inducible costimulatory molecule ligand
IFN-DC: DC subset differentiated with IFN-α
LAA: Leukemia-associated antigens
MDS: Myelodysplastic syndromes
MDSCs: Myeloid-derived suppressor cells
MHC: Major histocompatibility complex
MLC: Mixed-lymphocyte culture
Mo-DC: Monocyte-derived dendritic cells
NSCLC: Non-small cell lung cancer
ORR: Objective response rates
OS: Overall survival
PD-1: Programmed death 1
pDCs: Plasmacytoid DCs
PD-L1: Programmed death-ligand 1
PR: Partial remission
PRAME: Preferentially expressed antigen of melanoma
PS: Phosphatidylserine
TAA: Tumor-associated antigen
Tcms: Central memory T cells
Tems: Effector memory T cells
TIL: Tumor-infiltrating lymphocyte
TME: Tumor microenvironment
TSA: Tumor-specific antigens
WT1: Wilms’ tumor 1
